# Relation between ocular paraneoplastic syndromes and Immune Checkpoint Inhibitors (ICI): review of literature

**DOI:** 10.1186/s12348-023-00338-1

**Published:** 2023-04-06

**Authors:** Pauline Casselman, Julie Jacob, Pieter-Paul Schauwvlieghe

**Affiliations:** Department of Ophthalmology, University Hospitals Leuven, Herestraat 49, 3000 Leuven, Belgium

**Keywords:** Ocular paraneoplastic syndromes, Immune Checkpoint Inhibitors, Tumor response

## Abstract

**Purpose:**

To describe different ocular paraneoplastic syndromes in patients treated with Immune Checkpoint Inhibitors (ICI), its relation with different types of ICI and different types of tumors, and its implications for treatment.

**Methods:**

A comprehensive review of the literature was performed.

**Results:**

Patients treated with ICI can present with different ocular paraneoplastic syndromes, such as Carcinoma Associated Retinopathy (CAR), Melanoma Associated Retinopathy (MAR) and paraneoplastic Acute Exudative Polymorphous Vitelliform Maculopathy (pAEPVM). In literature, the different types of paraneoplastic retinopathy are mostly related to different types of primary tumors, with MAR and pAEPVM seen in melanoma, and CAR in carcinoma. Visual prognosis is limited in MAR and CAR.

**Conclusion:**

Paraneoplastic disorders result from an antitumor immune response against a shared autoantigen between the tumor and ocular tissue. ICI enhance the antitumor immune response, which can lead to increased cross-reaction against ocular structures and unmasking of a predisposed paraneoplastic syndrome. Different types of primary tumors are related to different cross-reactive antibodies. Therefore, the different types of paraneoplastic syndromes are related to different types of primary tumors and are probably unrelated to the type of ICI. ICI-related paraneoplastic syndromes often lead to an ethical dilemma. Continuation of ICI treatment can lead to irreversible visual loss in MAR and CAR. In these cases overall survival must be weighed against quality of life. In pAEPVM however, the vitelliform lesions can disappear with tumor control, which may involve continuation of ICI.

**Supplementary Information:**

The online version contains supplementary material available at 10.1186/s12348-023-00338-1.

## Background

Immune checkpoint inhibitors are considered a recent breakthrough in the treatment of advanced cancers [1]. The immune system contains several checkpoints to prevent overactivation against healthy cells. However, tumor cells use these checkpoints to escape the immune system. In some tumors, there is an upregulation of checkpoints on the T-cell surface, including cytotoxic T-lymphocyte antigen-4 (CTLA-4) receptor, and programmed death-1 (PD-1) receptor, thereby suppressing T-cell activation against tumor cells. Blocking this inhibitory interaction enhances a specific antitumor T-cell response.

To date, various PD-1 (pembrolizumab, nivolumab), PD-ligand-1 (PD-L1; atezolizumab), and CTLA-4 inhibitors (ipilimumab) have been approved in the treatment of several malignancies, including melanoma, non-small-cell lung carcinoma, and other advanced tumors.

The development of these new drugs has improved survival rates. However, immunotherapy removes a protection against autoimmunity allowing various immune-related adverse events (IRAE), with the most common being pneumonitis, hepatitis, colitis, dermatitis, and endocrinopathies [2, 3].

Ophthalmologic IRAE are rare and have been reported in less than 1% of patients [4–6]. Exact rates, however, are difficult to obtain. They typically develop within weeks to months of initiating therapy and can affect various parts of the eye and orbit. Most frequently reported ophthalmic adverse events include dry eye disease and uveitis (anterior uveitis, Vogt-Koyanagi-Harada disease-like uveitis). Other reported side effects are conjunctivitis, (peripheral ulcerative) keratitis, inflammatory orbitopathy, orbital myositis, myasthenia gravis, optic neuropathy, acute macular neuroretinopathy, and paraneoplastic syndromes, such as Carcinoma Associated Retinopathy (CAR), Melanoma Associated Retinopathy (MAR) and paraneoplastic Acute Exudative Polymorphous Vitelliform Maculopathy (pAEPVM).

Ocular paraneoplastic syndromes have been well described, but the evolution after treatment with ICI remains unclear. Therefore, we conducted a literature review to systematically map the research done in this area and identify existing gaps in knowledge. We focus mainly on its pathophysiology, clinical characteristics, diagnosis, and current treatment.

## Materials and methods

We performed a comprehensive literature search of the medical databases Medline (PubMed), and Embase, and Web of Science. The methodology of this literature review was written following the Preferred Reporting Items for Systematic Reviews and Meta-Analyses extension for Scoping Reviews (PRISMA-ScR) statement (Additional file 1). The search strategy is given in Additional file 2. To identify potentially relevant articles, two reviewers (PC and PPS) screened all search results based on the title and abstract. Selected full-text articles were then reviewed for eligibility. To avoid missing any relevant research, one reviewer (PC) performed snowballing, by which 24 additional articles were included. Two articles were found through hand searching. Additional file 3 provides a detailed overview of inclusion and exclusion criteria.

## Results

An overview of the available literature on this rare retinal manifestation is presented in Tables 1, 2 and 3.

**Table 1:** Cases of Melanoma Associated Retinopathy (MAR)

**Article**	**ICI**	**Age (y), gender**	**Previous therapy**	**Cancer**	**Symptoms**	**Initial BCVA**	**Time to onset**	**Antiretinal antibodies**	**Antitumor efficacy of ICI**	**Other IRAE**	**Treatment**	**Ophthalmological outcome**	**ICI discontinued**	**Recurrence after rechallenge**	**Follow-up**
Khaddour et al. (2021) [7]	Pembrolizumab	74, M	Surgery	Metastatic cutaneous melanoma	Nyctalopia and shimmering lights	NS	MAR before introduction of pembrolizumab	/	CR	Vitiligo	/	Significant improvement in visual symptoms, ERG normalization	After 17 cycles	No rechallenge	3.5 years
Poujade et al. (2021) [8]	Pembrolizumab	68, F	Surgery, ^a^	Metastatic conjunctival melanoma	Blurred vision, photosensitivity, floaters	NS	MAR before introduction of pembrolizumab	TRPM1	Regression of the gallbladder metastasis; increased vitiligo	NS	IVI DXM	Fewer floaters, but still undetectable dark-adapted ERG	N	No discontinuation	NS
Shahzad et al. (2021) [9]	Ipilimumab + nivolumab	56, M	Exenteration OS	Metastatic uveal melanoma	Flashing lights, visual aura	OD 6/18	3 weeks	NS	Partial response	Pneumonitis	Oral and intraocular CS^b^ IVI ranibizumab	Permanent loss of vision; macular scarring	Y	No rechallenge	Alive 22 months
Kim et al. (2020) [10]	Nivolumab	58, M	Surgery, radiotherapy	Metastatic cutaneous melanoma	Asymmetric vision loss	OD 20/32 OS 20/2000	4 cycles	TRPM1, aldolase C	NS	NS	IVI Bevacizumab OS x2 oral CS^c^	OD 20/50 OS hand motion SRF disappeared, PED remained	N	No discontinuation	4 months
Dolaghan et al. (2019) [11]	Ipilimumab/Nivolumab and pembrolizumab	72, M	NS	Metastatic melanoma	Bilateral uveitis	OS 6/24	2 cycles of I/N, 5 cycles of P	Recoverin, CA II	Complete radiological response	Grade 2 colitis, adrenal insufficiency and diabetes	Maxidex	Resolution of ocular inflammation, OS 6/24	Y	NS	NS
Elwood et al. (2021) [12]	Ipilimumab + nivolumab	65, F	Surgery	Metastatic malignant melanoma	Photopsia, visual field loss	OD 20/40 OS 20/50	4 cycles	60 kDa	Tumor regression without recurrence	Diarrhea, vomiting, and adrenal insufficiency	Bevacizumab OD, subtenon TA OS	OD 20/25 resolution of SRF and subretinal hyperreflectivity OS 20/100 Photopsia and VF improved	Y	No rechallenge	10 months
Kim et al. (2019) [13]	Nivolumab + ipilimumab	79, F	NS	Metastatic cutaneous melanoma	Floaters, photopsia, nyctalopia	OD 20/20 OS 20/25	1 cycle	Y, not in detail mentioned	CR	Transaminitis, rash, hypophysitis	IV CS, IVIG	20/20 OU, no improvement in DA	Y	No rechallenge	10 months
Roberts et al. (2016) [14]	Pembrolizumab	NS	Surgery, radiotherapy	Metastatic cutaneous melanoma	Nyctalopia, smoke-like vision	OD 20/20 OS 20/25	Shortly after initiation	23-kDa^*^, 30-kDa (CA II), 34-kDa, 40-kDa (aldolase), 42-kDa, 46-kDa (enolase), and 136-kDa	Partial response	NS	/	BCVA stable, gradual loss of pigmentation	N	No discontinuation	15 weeks
Audemard et al. (2013) [15]	Ipilimumab	70, F	Surgery, chemotherapy	Metastatic cutaneous melanoma	Progressive vision loss	NS	MAR before introduction of ipilimumab	NS	Stable disease	Vitiligo	CS before ipilimumab	BCVA decreased	N	No discontinuation	4 cycles

*ICI* Immune checkpoint inhibitor; y, years, *BCVA* Best corrected visual acuity, *IRAE* Immune-related adverse events, *M* Man, *NS* Not specified, *CR* Complete remission, *ERG* Electroretinography, *F* Female, *TRPM1* Transient receptor potential cation channel subfamily M member 1, *IVI* Intravitreal injection, *DXM* Dexamethasone, *N* No, *OS* Left eye, *OD* Right eye, *CS* Corticosteroids, *Y* Yes, *SRF* Subretinal fluid, *PED* Pigment epithelial detachment, CA II Carbonic anhydrase II, *TA* Triamcinolone acetonide, *VF* Visual field, *IV* Intravenous, *IVIG* Intravenous immunoglobulins, *OU* Both eyes, *DA* Dark adaptation

^a^Bilateral granulomatous anterior uveitis treated with corticoid eye drops; few months later vitritis/hyalitis (VKH-like) treated with oral corticosteroids; MAR treated with oral corticosteroid therapy (failed) and intravitreal dexamethasone injections (700 μg/injection) every 6 months (visual improvement)

^b^Pneumonitis treated with 50 mg prednisolone daily; MAR treated with 10 mg prednisolone daily, short-acting and long-acting intravitreal steroid implants (dexamethasone 0.7mg and fluocinolone acetonide, respectively)

^c^Oral prednisone 40mg daily, tapered over 1 week to 30mg four times daily; topical prednisolone acetate 1% four times daily in the right eye and twice daily in the left eye, cycloplegic drops; ketorolac tromethamine 0.5% 4 times daily OD; Ozurdex intravitreal implant OU

(*) not reactive to recoverin

**Table 2:** Cases of Carcinoma Associated Retinopathy (CAR)

**Article**	**ICI**	**Age (y), gender**	**Previous/concurrent therapy**	**Cancer**	**Symptoms**	**Initial BCVA**	**Time to onset**	**Antiretinal antibodies**	**Antitumor efficacy of ICI**	**Other IRAE**	**Treatment**	**ICI discontinued**	**Ophthalmological outcome**	**Recurrence after rechallenge**	**Follow-up**
Chauhan et al. (2022) [16]	Atezolizumab	75, M	C: chemotherapy	SCLC	Acute vision loss, floaters and photopsia	NLP	3 weeks	TULP1	NS	NS	Oral and local CS^a^; rituximab	Y	OD 20/40 OS 20/30 Fundus, OCT stable	N, rechallenge concomitant with CS and rituximab	27 weeks
Chen et al. (2022) [17]	Nivolumab	57, M	P: Chemotherapy	HCC	VF constriction OU	20/25 OU	16 days	Recoverin	NS	NS	Systemic CS^b^	Y	20/20 OU Slight VF improvement OCT, ERG almost stable	No rechallenge	24 months
Ghoraba et al. (2022) [18]	Pembrolizumab	52, F	P: surgery, radiotherapy, chemotherapy C: lenvatinib	Metastatic endometrial carcinoma	Nyctalopia, photosensitivity and photopsia	20/20 OU	18 months	CA II, enolase and arrestin	NS	Arrhythmia, electrolyte imbalance, hypothyroidism and severe diarrhea	/	Y	Full recovery of visual symptoms, normalization of full-field ERG	No rechallenge	7 months
Young et al. (2021) [19]	Nivolumab + Ipilimumab	NS	NS	Cervical cancer	NS	NS	NS	NS	NS	/	IV MP, plasmapheresis	NS	NS	NS	NS
Reddy et al. (2017) [20]	Nivolumab	64, F	P: chemotherapy, radiotherapy	Lung adenocarcinoma	Photopsias OD> OS, OD dark paracentral halo and color vision problems	OD 20/32 OS 20/20	Shortly afterwards	30-kDa (CA II), 35-kDa (GAPDH), 38-kDA, 58-kDa (PKM2), and 112-kDa	NS	Pericardial effusion, memory loss	PO CS^c^	Y	Resolution of symptoms, stability of ocular findings	No rechallenge	3 months

*ICI* Immune checkpoint inhibitor, *Y* Years, *BCVA* Best corrected visual acuity, *IRAE* Immune-related adverse events, *M* Man, *C* Concurrent, *SCLC* Small-cell lung cancer, *NLP* No light perception, *TULP1* Tubby-related protein 1, *NS* Not specified, *CS* Corticosteroids, *Y* Yes, *OD* Right eye, *OS* Left eye, *OCT* Optical coherence tomography, *N* No, *P* Previous, *HCC* Hepatocellular carcinoma, *VF* Visual field, *OU* Both eyes, *ERG* Electroretinography, *F* Female, *CA II* Carbonic anhydrase II, *IV* Intravenous, *MP* Methylprednisolone, *GAPDH* Glyceraldehyde-3-Phosphate Dehydrogenase, *PKM2* Pyruvate kinase M2, *PO* By mouth

^a^80 mg prednisone, tapered; 4 mg intravitreal preservative-free triamcinolone acetonide (Triesence, Alcon, Fort Worth, Texas, USA)

^b^Intravenous methylprednisolone (250 mg/day) for 3 days

^c^Oral prednisone 60 mg once daily; after 3 weeks slow taper of the prednisone, reducing by 10 mg every 3 weeks

**Table 3:** Cases of paraneoplastic Acute Exudative Polymorphous Vitelliform Maculopathy (pAEPVM)

**Article**	**ICI**	**Age (y), gender**	**Previous/concurrent/sequential therapy**	**Cancer**	**Symptoms**	**Initial BCVA**	**Time to onset**	**Antitumor efficacy of ICI**	**Other IRAE**	**Treatment**	**ICI discontinued**	**Ophthalmological outcome**	**Recurrence after rechallenge**	**Follow-up**
Lambert et al. (2021) [21]	Pembrolizumab	54, F	NS	Metastatic vaginal mucosal melanoma	OU: blurry vision OS: yellow spot in the central visual field	OU 20/20	4 cycles	Partial remission	Immune-related thyroiditis, sarcoid-like syndrome, grade 1 pneumonitis	/	Y	Total resolution of the subretinal fluid, vitelliform deposits persisted	No rechallenge	4 months
Kemels et al. (2020) [22]	Nivolumab	74, M	P: surgery S: Ipilimumab	Metastatic melanoma of the rectum	Visual loss	OD 20/25 OS 20/32	3 weeks	Metastatic progression	NS	PO CS^a^	Y	BCVA stable, mild decrease of subretinal fluid and stable subretinal material	No rechallenge	4 months (died)
Kemels et al. (2020) [22]	Nivolumab	51, F	/	Metastatic vulvovaginal mucosal melanoma	Decreased vision and light flashes	OD 20/32 OS 20/20	1 month	Progression Clinical significant reduction of metastatic lesions on CT scan 4 m after re-administration	Sarcoid-like granulomatous reaction	SC + IVI CS^b^	Y, after 6 cycles	No improvement	Rechallenge after 2 months with surgical resection of primary tumor → significant reduction of the SRF OU 20/25	10 months
Miyamoto et al. (2020) [23]	Nivolumab	73, M	/	Malignant nasal melanoma	Metamorphopsia OU	OD 20/20 OS 20/16	2 months	NS	NS	/	N	Fundoscopy worsened	No discontinuation	3 months, died 5 months after initial presentation
Miyakubo et al. (2019) [24]	Ipilimumab	78, M	P: surgery, chemotherapy; nivolumab x47	Metastatic cutaneous melanoma	VF impairment	OU 16/20	2 cycles	NS	NS	/	N	SRF initially increased and then decreased VF, BCVA stable	No discontinuation	> 3 months
Sandhu et al. (2019) [25]	Pembrolizumab	55, F	P: Dabrafenib, Trametinib, ipilimumab C: Vemurafenib, Dabrafenib	Metastatic cutaneous melanoma	OU: blurred vision	Initially normal, OU 20/40 3 weeks after the introduction of vemurafenib	5 days after starting Vemurafenib 3 months after starting Pembrolizumab	NS	NS	Difluprednate 4dd, dorzolamide 2dd	Vemurafenib discontinued	Gradual resolution of SRF OCT: normalization of the central retinal architecture with only a small foveal elevation above residual hyperreflective material OD 20/20 OS 20/25	No discontinuation	4 months (died 5 months after presentation)
Lincoff et al. (2016) [26]	Ipilimumab	65, M	/	Metastatic melanoma of gallbladder	Missing characters to the left of fixation for seconds at a time while reading	OD 20/25 OS 20/20	AEPVM before introduction of Ipilimumab	No signs of recurrence	NS	Ipilimumab and surgery	N	Symptoms slowly improved	No discontinuation	> 1 year
Crews et al. (2015) [27]	Ipilimumab	46, M	Surgery, radiotherapy	Metastatic cutaneous melanoma	Blurred vision and photophobia	20/100 OU	3 cycles	NS	NS	Elevated liver transaminases	IV CS^d^	OD 20/60 OS 20/40 at 1 week follow-up OCT: OD resolution of SRF, OS improvement of SRF	Y	No rechallenge
Mantopoulos et al. (2015) [28]	Ipilimumab	Early 70s, F	P: surgery	Acral lentiginous melanoma	OU: decreased vision, mild photophobia, ocular tenderness on palpation	OU 20/40	28 weeks	Progression	NS	Temozolomide and topical CS After 1 month: PO CS^c^	Y	OU 20/25 Resolution of SRF OCT: hyperreflective subretinal material	No rechallenge	> 6 months

*ICI* Immune checkpoint inhibitor, *Y* Years, *BCVA* Best corrected visual acuity, *IRAE* Immune-related adverse events, *F* Female, *NS* Not specified, *OU* Both eyes, *OS* Left eye, *Y* Yes, *M* Man, *P* Previous, *S* Sequential, *OD* Right eye, *PO* By mouth, *CS* Corticosteroids, *SC* Subconjunctival, *IVI* Intravitreal injection, *SRF* Subretinal fluid, *N* no, *VF* Visual field, *C* Concomitant, *OCT* Optical coherence tomography

Note: Column "autoimmune antibodies" was omitted as this was not studied in any case

^a^Methylprednisolone 64mg in tapering dose over 5 weeks

^b^Subconjunctival betamethasone, subconjunctival triamcinolone acetonide and an IVI with Ozurdex®

^c^Dexamethasone by mouth 4mg daily

^d^Intravenous dexamethasone 10 mg four times daily for 3 days

### MAR

We found nine cases of MAR related to ICI administration: 3 patients received the combination of ipilimumab and nivolumab, 3 pembrolizumab, 1 ipilimumab, 1 nivolumab, and 1 ipilimumab + nivolumab + pembrolizumab [7–15, 11, 12]. The mean age was 67.75 years (range 56 - 79), and there was an equal gender ratio (1 patient not specified (NS)). All patients had known metastatic melanoma with a history of surgery in 7 out of 9 (1 with radiotherapy and dacarbazine); in 2 patients any previous treatment was not reported. The three most frequently described presenting symptoms include visual impairment, photopsia and nyctalopia. Mean best corrected visual acuity (BCVA) at presentation was 20/35 (3 NS). Time to onset varied from a few days to a maximum of 5 cycles and in 3 cases MAR was already present before the start of ICI. In 5 cases, antiretinal antibodies were found with TRPM1, aldolase and carbonic anhydrase II (CA II) as the 3 most frequent.

The antitumor efficacy of ICI was a complete response in 37.5% (3/8), a partial response in 50% (4/8), and stable disease in 1 case (1 NS). Other IRAE occurred in 6 of 9 patients. MAR was treated with corticosteroids in 7 of 9 patients (3 systemic, 2 intraocular, 1 topical and 1 subtenon), 3 patients also received an intravitreal injection of anti-vascular endothelial growth factor to treat Macular Neovascularization (MNV). One patient received intravenous immunoglobulins (IVIG) in addition to corticosteroids. In 4 of 8 cases ICI was discontinued, but in none of the cases there was a rechallenge. BCVA was reported as an ophthalmic outcome in 7 cases (worse in 3, stable in 3, and better in one eye but worse in the other eye in 1 case). Improvement was seen in an eye with MNV. Inflammation resolved under corticosteroids. The mean follow-up was 56.1 weeks (range 3 - 182).

### CAR

Five CAR cases have been described: 2 with nivolumab, 1 with atezolizumab, 1 with pembrolizumab and one with the combination of nivolumab and ipilimumab [16–20]. There was an equal male-female ratio and the mean age was 62 years (range 52 - 75 years, 1 case age and gender NS). CAR was associated with lung carcinoma (*n=*2), hepatocellular carcinoma (*n=*1), cervical carcinoma (*n=*1) and endometrial carcinoma (*n=*1). One patient received chemotherapy concurrently and 1 lenvatinib (protein kinase inhibitor), 3 patients had already been treated before the start of ICI (1 chemotherapy, 1 chemotherapy + radiotherapy, and 1 surgery + radiotherapy + chemotherapy; 1 NS). Photopsias are the most frequently reported symptoms (*n=*3; 1 NS) and mean BCVA at presentation was 20/60 (1 NS, range No Light Perception-20/20). Time to onset was shortly afterwards (3 weeks, 2 cycles and “shortly thereafter”) in 3 patients, 18 months in 1 patient and was not reported in 1 case. Antiretinal antibodies were detected in 4 patients (CA II (*n=*2), TULP1, recoverin, GAPDH, 38 kDa, PKM2, 112 kDa, enolase and arrestin). The antitumor efficacy of ICI has not been discussed in any article. In 2 cases other IRAE occurred (arrhythmia, electrolyte imbalance, hypothyroidism, diarrhea, pericardial effusion, and memory loss).

In 3 of 5 patients CAR was treated with systemic corticosteroids, in 1 case this was in combination with rituximab. In all patients there was an improvement in presenting complaints (*n=*4; 1 NS). Visual acuity remained stable or improved in all cases (1 NS). In 4 patients ICI was discontinued (1 NS) and in 1 patient rechallenge together with corticosteroids and rituximab did not lead to a recurrence. The mean duration of follow-up was 9.25 months (range 3 - 24 months, 1 NS).

### pAEPVM

The search strategy yielded nine cases of pAEPVM related to ICI (ipilimumab *n=*4, nivolumab *n=*3, pembrolizumab *n=*2) [21–28]. The mean age was 62.8 years (range 46 - 78). 5 of 9 patients were male. The primary tumor in all cases was a melanoma, mainly mucocutaneous. In 2 patients the tumor had already been treated surgically, 1 had a history of surgery and radiotherapy, and 1 of surgery, chemotherapy and nivolumab. The patient described by Sandhu et al. was previously treated with a B-type Raf proto-oncogene (BRAF) inhibitor, Mitogen-activated protein kinase kinase (MEK) inhibitor and ipilimumab. Pembrolizumab was given concomitantly with 2 BRAF inhibitors, dabrafenib and vemurafenib. 1 patient received ipilimumab after nivolumab. In 3 patients, ICI was the first-line treatment (1 patient NS). Mild loss of vision is the most frequently described symptom, reported in 6 out of 9 patients. BCVA at presentation was 20/25 (range 20/100 - 20/20). Time to onset averaged 10.25 weeks (range 3-28). The antitumor efficacy of ICI was discussed in 5 of the 9 cases and varied widely: progression (*n=*2), partial remission (*n=*1), reduction after rechallenge (*n=*1), and no recurrence (*n=*1). Other IRAE occurred in 33% (immune-related thyroiditis, sarcoid-like syndrome, elevated liver transaminases, and pneumonitis). The ICI was stopped in 5 patients and in the case of Sandhu et al.. Vemurafenib (BRAF inhibitor) was stopped. In 3 cases no additional treatment was started, and 5 patients received corticosteroids (systemic (*n=*2), intraocular, topical and in 1 case together with chemotherapy). In the case of Lincoff et al., pAEPVM was already present before the start of ipilimumab. After surgery and initiation of this ICI, a slow improvement in symptoms occurred. Only in 1 of 5 patients (Kemels et al.) a rechallenge occurred together with surgical resection of the primary tumor, after which a significant reduction of the subretinal fluid (SRF) was noted. In most cases there was a resolution of the SRF (*n=*6), and the subretinal deposits (*n=*3) persisted. The mean duration of follow-up was 17.4 weeks (range 3-40).

## Discussion

We describe the findings of ocular paraneoplastic syndromes with checkpoint inhibitors. A comparison between the three paraneoplastic syndromes is presented in Table 4.

**Table 4:** Characteristics of paraneoplastic syndromes

	**MAR**	**CAR**	**pAEPVM**
**Autoantibodies against**	Bipolar cells	Photoreceptors	RPE cells
**Associated tumor**	Melanoma	SCLC	Mucosal melanoma
**Antibody testing**	TRPM1, recoverin, α-enolase, CA II	Recoverin, α -enolase, CA II	Bestrophin, usually not performed
**Symptoms**	Very symptomatic: progressive painless visual loss, photopsias, nyctalopia, shimmering, visual field defects, light sensitivity in case of inflammation		Few symptoms: blurred vision, metamorphopsia, nyctalopia, and photopsias
**Retinal signs**	Initially normal or subtle: retinal vessel attenuation, optic disc pallor, vitreous cells, pigmentary changes		More prominent: multifocal serous retinal detachments and subretinal deposits with a vitelliform appearance in the posterior pole
**OCT**	Non-specific	Loss of the outer retinal layers with foveal sparing	Subretinal fluid and deposits of hyperreflective material
**FA**	Non-specific	Non-specific, sometimes retinal vasculitis	Blockage at the vitelliform lesion without retinal or papillary leakage
**FAF**	Non-specific	Hyperautofluorescence around a hypoautofluorescent zone	Hyperautofluorescence corresponding to the deposits
**VF**	(para)central scotoma, peripheral constriction		Non-specific
**ERG**	Electronegative	Extinguished rod and cone responses, mainly affecting the rods	Normal
**Treatment**	- Corticosteroids in case of inflammation - Interruption of ICI? - Tumor control - Rituximab/IVIG/plasmapheresis/efgartigimod?		- Corticosteroids in case of inflammation - Tumor control - Wait and see
**Prognosis**	Poor	Poor	Usually good

*MAR* Melanoma associated retinopathy, *CAR* Carcinoma associated retinopathy, *pAEPVM* Paraneoplastic Acute Exudative Polymorphous Vitelliform Maculopathy, *RPE* Retinal pigment epithelium, *SCLC* Small-cell lung carcinoma, *TRPM1* Transient receptor potential cation channel subfamily M member 1, *CA II* Carbonic anhydrase II, *OCT* Optical coherence tomography, *FA* ​​Fluorescein angiography, *FAF* Fundus autofluorescence, *VF* Visual field, *ERG* Electroretinography, *ICI* Immune checkpoint inhibitors, *IVIG* Intravenous immunoglobulins

Interestingly, these paraneoplastic syndromes are mainly seen in specific primary tumors. For example, MAR is exclusively described in melanomas; CAR mainly in patients with small cell lung carcinoma, but is associated with a variety of cancers. pAEPVM has also been documented in several melanoma and carcinoma cases, but is often related to mucosal melanoma. However, ICI were initially only indicated in metastatic melanoma, which may skew these results.

MAR, CAR and pAEPVM are rare retinopathies that can occur without or after initiation of ICIs. Given that only case reports exist for now, the exact incidence of these paraneoplastic syndromes whether or not in association with ICIs is currently unknown. Since ICIs can induce an increased anti-tumor response, a potential cross-reaction may result in exacerbation or induction of a predisposed paraneoplastic phenomenon.

The exact underlying pathophysiology is not yet fully understood, but molecular mimicry is the globally accepted mechanism. Presumably, the increased anti-tumor response induced by ICI leads to an increased cross-reaction of antibodies against non-tumor antigens; namely against the Retinal Pigment Epithelium (RPE) in pAEPVM, against bipolar cells in MAR, and against photoreceptors in CAR [15, 22, 23, 29–32].

Antiretinal autoantibodies give rise to bilateral retinal damage and visual disturbances, which are much more pronounced in CAR and MAR compared to pAEPVM [26, 30, 32, 33]. In CAR, cone dysfunction results in a decrease in visual acuity, impaired color vision, and central scotomas. A dysfunction of the rods is more likely to lead to prolonged dark adaptation, nyctalopia and (mid)peripheral visual field defects/scotomas.

The time to onset varies between 2 weeks and 18 months.

In pAEPVM, the antibodies probably directed against RPE, disrupt their pump and transport function. It is believed to be an immune response against bestrophin [34, 35]. The clinical picture therefore resembles autosomal recessive bestrophinopathy with the only difference that the latter has a shallow anterior chamber. Subretinal fluid and subretinal accumulation of yellowish material occurs at the posterior pole [21, 22, 25]. These vitelliform lesions are typically hyperautofluorescent indicating lipofuscin deposition in the RPE cells [36]. Optical coherence tomography (OCT) shows zones of subretinal fluid and deposits of hyperreflective material. Fluorescein angiography (FA) reveals blockage at the vitelliform lesion without retinal or optic nerve leakage.

In CAR and MAR fundoscopic findings are initially rather subtle with sometimes retinal vessel attenuation, and presence of intraocular inflammation; evolving into retinal pigment epithelial mottling, retinal atrophy, and optic disc pallor [30, 33, 37]. OCT shows loss of the outer retinal layers with foveal sparing. A (para)central scotoma can be visualized on the visual field. Findings on fundus autofluorescence (FAF) and FA are rather variable and not pathognomonic. In CAR, FA sometimes shows retinal vasculitis. Hyperautofluorescence around a hypoautofluorescent zone reflects the actively affected photoreceptors in CAR.

Full-field electroretinography (ERG) provides an objective evaluation of retinal function and is therefore an important diagnostic test [33]. In CAR, depending on the degree of damage to the rods and/or cones, a reduction of the a-wave and consequently b-wave is seen, most pronounced in photopic and/or scotopic conditions. In MAR, ERG reflects impaired ON-bipolar cell function which typically manifests as an electronegative ERG. This pattern is also seen in the complete type of congenital stationary night blindness (cCSNB) [38].

In pAEPVM, a normal ERG is seen.

In addition to its diagnostic value, ERG can also be considered as an indicator of treatment response.

Antibody testing, detected by Western blot, enzyme-linked immunosorbent assay, or immunohistochemical methods, is another interesting diagnostic tool [33]. Numerous antiretinal antibodies have been characterized in CAR and MAR [39, 40]. The most commonly described antiretinal antibodies include recoverin, a 23 kDa calcium binding protein found on photoreceptors; α -enolase, a 46 kDa ubiquitous glycolytic enzyme; arrestin (48 kDa), CA II (30 kDa), and transient receptor potential cation channel subfamily M member 1 (TRPM1) expressed on retinal ON bipolar cells. TRPM1 mediates its depolarization in response to light, which is reflected in the b-wave on ERG. Mutations in the TRPM1 gene have also been documented in CSNB [41, 42]. In autoimmune retinopathy the seropositivity for known antiretinal antibodies at presentation is only 50 - 65% [43–45]. In addition, antiretinal antibodies can also be found in control patients. The absence of antiretinal antibodies therefore does not exclude the diagnosis.

Given the progressive visual impairment especially in CAR and MAR, rapid diagnosis and early treatment initiation is crucial. However, the treatment of ocular paraneoplastic syndromes can be challenging. Many treatment options have been described in literature, but globally there are two strategies [15]. On the one hand, reduction of autoimmunity can be achieved through immunosuppression or immunomodulation. On the other hand, tumor cytoreduction, obtained by surgery, chemotherapy or immunotherapy, can lead to decreased tumor antigen production and thus decreased cross-reaction [46].

With better tumor control by resection of the primary tumor or good effect of ICI, the tumor load can be reduced or disappear, resulting in a reduced T-cell and secondary decreased B-cell response with consequently less cross-reaction [30]. Hence, sometimes, improvement can occur after using ICI as described in some articles [7].

Strikingly, paraneoplastic syndromes might be associated with a favorable tumor response in metastatic melanoma [47].

On the other hand, it is sometimes difficult to wait for the beneficial effect, because damage can occur fairly quickly, especially with CAR and MAR. This damage is irreversible, even after tumor control. In those cases, it may be indicated to stop the ICI and still try corticosteroids and/or other immunosuppressive/immunomodulatory therapy. Since there is no pronounced decrease in vision with pAEPVM, a wait-and-see approach can be considered [22].

Suppression of autoimmunity can be achieved through multiple mechanisms, such as corticosteroids, rituximab, IVIG, and plasmapheresis; however, there is conflicting evidence in literature, with varying degrees of success [30, 46].

Tapering dose systemic corticosteroids are also sometimes administered. However, the potential negative impact of this drug on tumor response should be taken into account when used before or in conjunction with ICI [48]. Therefore, this decision is always made in consultation with an oncologist.

Ideally, the treatment provides good tumor control, resulting in less cross-reactivity. Furthermore, the Ig(immunoglobulin)-mediated side effects should be tackled, without compromising tumor response.

Novel immunotherapeutic drugs, such as efgartigimod or rozanolixizumab aim at reducing pathogenic autoantibodies by inhibiting the neonatal Fc receptor (FcRn) for binding immunoglobulin G (IgG) [49]. These drugs have a high affinity for FcRn and compete with IgG to bind this receptor. Since FcRn protects IgGs against lysosomal degradation and thereby prolongs their half-life, these drugs can reduce circulating IgG antibodies. These new drugs target pathological IgG and thus may act specifically on humoral immunity while not affecting cellular T cell immunity which is important for tumor control. This may show promise in paraneoplastic exacerbations after ICI.

Based on the pathophysiology, pAEPVM is known to be reversible, which explains its relatively favorable visual prognosis. This is in contrast to CAR and MAR where the damage at the level of the photoreceptors or bipolar cells is irreversible, resulting in a poor visual prognosis. This is in line with the included case reports in which a fairly good visual outcome is described for pAEPVM, in contrast to CAR and MAR.

## Conclusion

Immune checkpoint inhibitors can induce an exacerbation of paraneoplastic syndromes via an increased antitumor response and thus cross-reaction against ocular structures, among others. The type of paraneoplastic syndrome varies by tumor. The diagnosis is mainly clinical, in which electroretinography and determination of serum antiretinal autoantibodies offer a diagnostic added value, especially for CAR and MAR. The treatment remains controversial where good tumor control is desired with consequent reduction of cross-reactivity, combined with suppression of immunoglobulin-associated side effects.

## Supplementary Information

**Additional file 1.**

**Additional file 2. **

**Additional file 3.**

## Data Availability

Data sharing not applicable to this article as no datasets were generated or analyzed during the current study.

## Abbreviations

BCVA: Best corrected visual acuity
BRAF: B-type Raf proto-oncogene
CA II: Carbonic anhydrase II
CAR: Carcinoma Associated Retinopathy
cCSNB: Complete type of congenital stationary night blindness
CTLA-4: Cytotoxic T-lymphocyte antigen-4 receptor
ERG: Electroretinography
FA: Fluorescein angiography
FAF: Fundus autofluorescence
FcRn: Neonatal FC receptor
ICI: Immune checkpoint inhibitors
Ig: Immunoglobulin
IRAEs: Immune-related adverse events
IVIG: Intravenous immunoglobulins
MAR: Melanoma Associated Retinopathy
MEK: Mitogen-activated protein kinase kinase
MNV: Macular Neovascularization
NS: Not specified
OCT: Optical coherence tomography
pAEPVM: Paraneoplastic Acute Exudative Polymorphous Vitelliform Maculopathy
PD-1: Programmed death-1 receptor
PD-L1: Programmed death-ligand-1
PRISMA-ScR: Preferred Reporting Items for Systematic Reviews and Meta-Analyses extension for Scoping Reviews
RPE: Retinal pigment epithelium
SRF: Subretinal fluid
TRPM1: Transient receptor potential cation channel subfamily M member 1
