# Supplementary material for: Relation between ocular paraneoplastic syndromes and Immune Checkpoint Inhibitors (ICI): review of literature
Source: J Ophthalmic Inflamm Infect. 2023 Apr 6;13:16. doi: 10.1186/s12348-023-00338-1 (PMC10079794; doi:10.1186/s12348-023-00338-1)
Supplement: Supplementary file 1 — Additional file 1. [file 12348_2023_338_MOESM1_ESM.docx]

**SEARCH STRATEGY**

**PubMed**

Concept 1: immune checkpoint inhibitors

"Immune Checkpoint Inhibitors"[Mesh] OR "Immune Checkpoint Inhibitor*"[tiab] OR "Immune Checkpoint Block*"[tiab] OR "Immune Checkpoint Inhibition"[tiab] OR "PD-L1 Inhibitor*"[tiab] OR "PD L1 Inhibitor*"[tiab] OR "Programmed Death-Ligand 1 Inhibitor*"[tiab] OR "Programmed Death Ligand 1 Inhibitor*"[tiab] OR "PD-1-PD-L1 Blockade"[tiab] OR "PD 1 PD L1 Blockade"[tiab] OR "CTLA-4 Inhibitor*"[tiab] OR "CTLA 4 Inhibitor*"[tiab] OR "Cytotoxic T-Lymphocyte-Associated Protein 4 Inhibitor*"[tiab] OR "Cytotoxic T Lymphocyte Associated Protein 4 Inhibitor*"[tiab] OR "PD-1 Inhibitor*"[tiab] OR "PD 1 Inhibitor*"[tiab] OR "Programmed Cell Death Protein 1 Inhibitor*"[tiab] OR "Ipilimumab"[tiab] OR "Tremelimumab"[tiab] OR "Pembrolizumab"[tiab] OR "Nivolumab"[tiab] OR "Cemiplimab"[tiab] OR "Atezolizumab"[tiab] OR "Avelumab"[tiab] OR "Durvalumab"[tiab]

Concept 2: ocular paraneoplastic syndromes

"Paraneoplastic Syndromes, Ocular"[Mesh] OR "Ocular Paraneoplastic Syndrome*"[tiab] OR "Ocular Paraneoplastic Disease*"[tiab] OR "Melanoma-Associated Retinopath*"[tiab] OR "Melanoma Associated Retinopath*"[tiab] OR "Paraneoplastic Melanocytic Proliferation*"[tiab] OR "Cancer-Associated Retinopath*"[tiab] OR "Cancer Associated Retinopath*"[tiab] OR “Acute Exudative Polymorphous Vitelliform Maculopathy"[tiab]

**Embase**

Concept 1: immune checkpoint inhibitors

'immune checkpoint inhibitor'/exp OR 'immune checkpoint inhibitor*':ti,ab,kw OR 'immune checkpoint block*':ti,ab,kw OR ‘Immune Checkpoint Inhibition’:ti,ab,kw OR ‘PD-L1 Inhibitor*’:ti,ab,kw OR ‘PD L1 Inhibitor*’:ti,ab,kw OR ‘Programmed Death-Ligand 1 Inhibitor*’:ti,ab,kw OR ‘Programmed Death Ligand 1 Inhibitor*’:ti,ab,kw OR ‘PD-1-PD-L1 Blockade’:ti,ab,kw OR ‘PD 1 PD L1 Blockade’:ti,ab,kw OR ‘CTLA-4 Inhibitor*’:ti,ab,kw OR ‘CTLA 4 Inhibitor*’:ti,ab,kw OR ‘Cytotoxic T-Lymphocyte-Associated Protein 4 Inhibitor*’:ti,ab,kw OR ‘Cytotoxic T Lymphocyte Associated Protein 4 Inhibitor*’:ti,ab,kw OR ‘PD-1 Inhibitor*’:ti,ab,kw OR ‘PD 1 Inhibitor*’:ti,ab,kw OR ‘Programmed Cell Death Protein 1 Inhibitor*’:ti,ab,kw OR ‘Ipilimumab’:ti,ab,kw OR ‘Tremelimumab’:ti,ab,kw OR ‘Pembrolizumab’:ti,ab,kw OR ‘Nivolumab’:ti,ab,kw OR ‘Cemiplimab’:ti,ab,kw OR ‘Atezolizumab’:ti,ab,kw OR ‘Avelumab’:ti,ab,kw OR ‘Durvalumab’:ti,ab,kw

Concept 2: ocular paraneoplastic syndromes

'ocular paraneoplastic syndrome'/exp OR 'ocular paraneoplastic syndrome*':ti,ab,kw OR ‘Ocular Paraneoplastic Disease*’:ti,ab,kw OR ‘Melanoma-Associated Retinopath*’:ti,ab,kw OR ‘Melanoma Associated Retinopath*’:ti,ab,kw OR ‘Paraneoplastic Melanocytic Proliferation*’:ti,ab,kw OR ‘Cancer-Associated Retinopath*’:ti,ab,kw OR ‘Cancer Associated Retinopath*’:ti,ab,kw OR ‘Acute Exudative Polymorphous Vitelliform Maculopathy’:ti,ab,kw

**Web of Science**

Concept 1: immune checkpoint inhibitors

"Immune Checkpoint Inhibitors" OR "Immune Checkpoint Inhibitor*"OR "Immune Checkpoint Block*"OR "Immune Checkpoint Inhibition"OR "PD-L1 Inhibitor*"OR "PD L1 Inhibitor*"OR "Programmed Death-Ligand 1 Inhibitor*"OR "Programmed Death Ligand 1 Inhibitor*"OR "PD-1-PD-L1 Blockade"OR "PD 1 PD L1 Blockade"OR "CTLA-4 Inhibitor*"OR "CTLA 4 Inhibitor*"OR "Cytotoxic T-Lymphocyte-Associated Protein 4 Inhibitor*"OR "Cytotoxic T Lymphocyte Associated Protein 4 Inhibitor*"OR "PD-1 Inhibitor*"OR "PD 1 Inhibitor*"OR "Programmed Cell Death Protein 1 Inhibitor*"OR "Ipilimumab"OR "Tremelimumab"OR "Pembrolizumab"OR "Nivolumab"OR "Cemiplimab"OR "Atezolizumab"OR "Avelumab"OR "Durvalumab"[tiab]

Concept 2: paraneoplastic syndromes

"Paraneoplastic Syndromes, Ocular" OR "Ocular Paraneoplastic Syndrome*"OR "Ocular Paraneoplastic Disease*"OR "Melanoma-Associated Retinopath*"OR "Melanoma Associated Retinopath*"OR "Paraneoplastic Melanocytic Proliferation*"OR "Cancer-Associated Retinopath*"OR "Cancer Associated Retinopath*"OR “Acute Exudative Polymorphous Vitelliform Maculopathy"
