# Supplementary material for: Relation between ocular paraneoplastic syndromes and Immune Checkpoint Inhibitors (ICI): review of literature
Source: J Ophthalmic Inflamm Infect. 2023 Apr 6;13:16. doi: 10.1186/s12348-023-00338-1 (PMC10079794; doi:10.1186/s12348-023-00338-1)
Supplement: Supplementary file 2 — Additional file 2. [file 12348_2023_338_MOESM2_ESM.docx]

Additional records identified through other sources

-Snowballing (n = 24)

-Hand searching (n = 2)

Records identified through searching multiple databases (n = 45)

**Identification**

Removal of duplicates (n = 18)

**Screening**

Records excluded. (n = 3)

Records screened by title and abstract (n = 53)

Full-text articles excluded, with reason no full-text (n = 1)

Full-text articles assessed for eligibility (n = 50)

**Eligibility**

Articles included in scoping review (n = 49)

**Included**

**Additional file 2:** Flowchart of the systematic search and selection process following the Prisma statement.
