# Supplementary material for: Relation between ocular paraneoplastic syndromes and Immune Checkpoint Inhibitors (ICI): review of literature
Source: J Ophthalmic Inflamm Infect. 2023 Apr 6;13:16. doi: 10.1186/s12348-023-00338-1 (PMC10079794; doi:10.1186/s12348-023-00338-1)
Supplement: Supplementary file 3 — Additional file 3. [file 12348_2023_338_MOESM3_ESM.docx]

| **Inclusion criteria** | **Exclusion criteria** |
| --- | --- |
| - Included cancer patients with MAR, CAR or pAEPVM diagnosed before or after treatment with ICIs - Described the occurrence of autoimmune retinopathy in association with immune checkpoint inhibitors - Original publications in English - Full-text available | - Not related to outcomes of interest - Language other than English - No full-text available |

**Additional file 3:** Detailed eligibility criteria.

Abbreviations: MAR, melanoma associated retinopathy; CAR, carcinoma associated retinopathy; pAEPVM, paraneoplastic Acute Exudative Polymorphous Vitelliform Maculopathy; ICIs, immune checkpoint inhibitors
